# Lightweight and Resource-Constrained Learning Network for Face Recognition with Performance Optimization

**DOI:** 10.3390/s20216114

**Published:** 2020-10-27

**Authors:** Hsiao-Chi Li, Zong-Yue Deng, Hsin-Han Chiang

**Affiliations:** 1 Department of Computer Science and Information Engineering, Fu Jen Catholic University, No. 510, Zhongzheng Road, Xinzhuang District, New Taipei City 242, Taiwan; hcli@csie.fju.edu.tw; 2 Department of Electrical Engineering, National Taiwan Normal University, No 162, Sec. 1, Heping East Road, Da’an District, Taipei City 106, Taiwan; yondeng@gmail.com

**Keywords:** face recognition, deep convolutional network, resource constraints, lightweight optimization, surveillance system, FaceNet

## Abstract

Despite considerable progress in face recognition technology in recent years, deep learning (DL) and convolutional neural networks (CNN) have revealed commendable recognition effects with the advent of artificial intelligence and big data. FaceNet was presented in 2015 and is able to significantly improve the accuracy of face recognition, while also being powerfully built to counteract several common issues, such as occlusion, blur, illumination change, and different angles of head pose. However, not all hardware can sustain the heavy computing load in the execution of the FaceNet model. In applications in the security industry, lightweight and efficient face recognition are two key points for facilitating the deployment of DL and CNN models directly in field devices, due to their limited edge computing capability and low equipment cost. To this end, this paper provides a lightweight learning network improved from FaceNet, which is called FN13, to break through the hardware limitation of constrained computational resources. The proposed FN13 takes the advantage of center loss to reduce the variations of the between-class features and enlarge the difference of the within-class features, instead of the triplet loss by using FaceNet. The resulting model reduces the number of parameters and maintains a high degree of accuracy, only requiring few grayscale reference images per subject. The validity of FN13 is demonstrated by conducting experiments on the Labeled Faces in the Wild (LFW) dataset, as well as an analytical discussion regarding specific disguise problems.

## 1. Introduction

Despite recent advances in image sensor technology, cameras have always played an important role in security. In most cases, cameras are usually installed in a fixed position, but in order to realize smart monitoring systems, a highly accurate embedded system and a large computing device that can work properly under low power consumption circumstances are needed. In recent decades, the development of science and technology has advanced rapidly, and large demands have arisen in the surveillance industry [1]. As a result, multi-camera systems have become widely used for surveillance and access control under various situations in the hopes of deterring crimes and improving both public and private security. Relying on human beings to monitor the views is unrealistic and usually insufficient since it typically happens when requested. As such, face recognition in automatic identification can be considered a non-intrusive and relatively reliable method for user authentication by using their face. Furthermore, due to the emergence of deep learning methodologies and the advance of hardware, it has become feasible to perform automatic face recognition on high-end processors easily and with none of the aforementioned limitations. Recently, deep feature extraction approaches on the basis of deep learning, especially the convolutional neural network (CNN), have revealed remarkable advantages and in-depth development in face recognition technology. Among them, FaceNet [2], proposed by Google researchers in 2015, is one of the representative methods based on the deep learning architecture of CNN in the literature.

**Figure 1:**
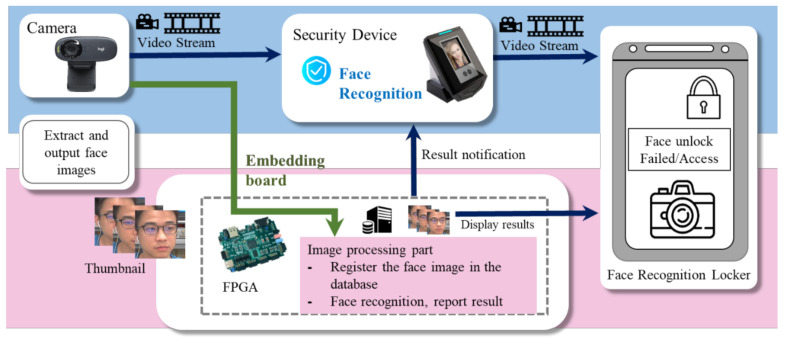
The architecture of a typical face recognition embedded system on a security device.

Nowadays, computing devices using human face recognition are being increasingly applied under various contexts, such as secure access and time attendance systems. Figure 1 depicts the architecture of a typical embedded face recognition system on a security device. In some common scenarios, the computing device with user-friendly face recognition may store one or more enrollment facial images of the authorized users. While a face is presented to the camera of a security device with face recognition enabled, the captured image is then converted into mathematical data by the algorithm. Thereafter, the unique mathematic representation, as extracted from the face image of a user, is compared to the underlying face database. If the probed facial features of the user match the stored facial features in terms of a high matching score, user access will be granted, and shift data for recording attendance can also be collected in an effective way. So far, since deep learning (DL) with CNN is becoming the leading technology and a promising prospect for a wide range of applications, face recognition relying on an embedded platform with limited onboard resources, such as processors, memory, and batteries, is in urgent demand for deployment in embedded systems [3].

In order to make automatic camera surveillance or access control systems available with the aid of face recognition, many extended devices with high computing power in the CPU or even the Graphical Processing Unit (GPU) have been successfully applied to perform recognition and verification tasks via deep learning networks. Although several deep convolutional networks have exhibited a promising accuracy of over 99% when executed on CPUs and GPUs, implementing such complex architectures directly in resource-constrained embedded devices is a great challenge. Due to attractive features such as higher throughput and energy efficiency, embedded devices with CNN acceleration are promising platforms for image processing and deep learning applications. However, deployment of DL and CNN in embedded devices still presents challenges mainly resulting from the limited hardware resources and strong demand for careful design and performance optimization [4]. Thus, a lightweight face recognition model, which can specifically be compliant to a resource-constrained development environment, is expected to make the automatic camera surveillance system more feasible on embedded devices.

The related works on face recognition are discussed in Section 2. In addition, the remainder of the work is organized as follows. Section 3 develops a lighter face recognition framework conceptualized on the basis of FaceNet. Section 4 presents the experiments with conducted results. In addition, further discussions are provided. The conclusions and future works are summarized in Section 5.

## 2. Related Works

Traditional face recognition is composed of three tasks: face detection, feature extraction, and face matching/face classification. To perform automatic face recognition, many methods have been proposed to represent faces or extract face features, such as principal component analysis (PCA) [5], Fisher’s discriminant analysis (FDA), linear discriminant analysis (LDA) [6], neural networks [7], scale-invariant feature transform (SIFT), discrete cosine transform (DCT), wavelet transform, and other feature extraction methods. Among them, PCA has been one of the most widely used techniques for extracting face features, as it can be simply applied in practical problems. PCA can reduce the dimensions of face features and easily perform the matching/classification task. However, PCA suffers from posture changes and variations in lighting. FDA and LDA are statistical approaches that attempt to maximize the distance between different identities and minimize the variance within samples for the same identity projected onto the face feature space. The first neural network applied to face recognition is a single layer network. This network distinguishes each identity by proposing a separate network. Other machine learning techniques are applied with a face being represented by the predefined feature expression, which characterizes a face through multiple processing modules. Once the feature is extracted, classifiers, such as Support Vector Machine (SVM) [8], Random Forest [9], and K-nearest neighbor (KNN) [10], can be applied to distinguish patterns of the sample images for the same person from those of different people. However, the extracted features influence the recognition result greatly.

Most recently, CNN was proposed to perform feature extraction and pattern classification in one multi-layer network. CNN takes the original image instead of the characteristic feature as input to learn the best feature representation automatically. As being the most representative method among many works of literature that apply the CNN variants for face recognition, FaceNet consists of a set of trained layers, known as face identifiers, and an intermediate bottleneck layer, representing generalized recognition beyond the set of identifiers. It converts face images into a *k*-dimensional feature space as face feature embedding, which is similar to word embedding. To discriminate the faces from one identity from those of other identities, FaceNet employs triplet loss for enforcing margin distances between each pair of faces. Therefore, similarity and difference can be calculated among various faces. The *k*-dimensional embedding technology established by this model can cluster faces effectively and accurately. With FaceNet-generated face embeddings as features, face recognition and verification can subsequently be performed. Similar images would result in a closer distance in the embedding space, while non-similar images would have their corresponding embeddings much further away from each other. Although FaceNet could achieve over 99% accuracy on LFW, it still faces the challenge of its large model size, which makes it unrealistic to implement in mobile environments or even in embedded devices. Furthermore, OpenFace [11] and some other models originating from FaceNet, but with smaller sizes, as stated in [12], have been designed for use in mobile applications. These models successfully reduced the model size by about 60%–80% (12.5 MB–30 MB) compared with FaceNet (90 MB); however, in the reduced versions, the error rate increased by up to 16 times and did not fit the common hardware limitations of embedded devices.

Other than FaceNet, many other CNNs have been applied and modified for different applications with face recognition as the foundation. Additionally, more and more facial databases have been created to reach the goal. Almabdy et al. [13] investigated the performance of a hybrid model with pre-trained CNN applied to perform feature extraction followed by linear-based SVMs to perform multi-class classification. In their research, two networks, AlexNet and ResNet-50, were constructed to extract facial features. Additionally, AlexNet was applied for transfer learning, with the network acting as both feature extractor and classifier, simultaneously. The results of the conducted experiment were then compared to the hybrid model. To make SVM learn the best discriminations among the subjects, multiple images were needed for every single identity to construct the networks. The results showed an accuracy of 94% on the LFW dataset for SVM in classifying the identities based on the network-extracted features. Besides, the transfer learning network using AlexNet achieved an accuracy of 95.63% on the LFW dataset. In another study proposed by Yang et al. [14], the CNN model for face recognition was improved by fusing the face features extracted via AlexNet with features extracted through processes of scale-invariant feature transformation (SIFT) and rotation-invariant texture features (RITF). In their study, Random Forest was used as a classifier for the fused features. According to their experiments, the enhanced fused features helped improve the model, with a true positive rate (TPR) increase of 10.97–13.24%, achieving an accuracy of 98.98% on the LFW dataset. Due to the introduction of SIFT-RITF features, the model’s computing time was greatly increased. Therefore, the study made use of a graphics processing unit (GPU) to reduce the computing time, reaching 5–6 times acceleration compared to Central Processing Unit (CPU)-based computing.

Cuculo et al. [15] proposed a method based on a VGG-face network. The authors took advantage of the augmentation process of face images to increase the recognition ability of their network which only accounts for one single reference image per subject. With a sparse sub-dictionary learning process, the model was able to derive a concise description for each face image. And the identity would be recognized via their *L*_0_-norm minimization algorithm with a majority voting optimizer. The results showed effectiveness on large datasets, with the model outperforming other state-of-the-art approaches on very low-resolution images and images with some disguises. Abdallah et al. [16] proposed a zero-shot learning model consisting of 19 CNN layers for person spotting and face clustering in video stream data. The proposed network extracts face feature vectors similar to FaceNet-extracted embeddings from the pre-whitening processed video frames. Prior to clustering, softmax loss was applied to calculate the face similarity among all feature vectors. New face clusters would be created if the similarity values did not match any existing clusters under a preset threshold. Their model outperformed conventional clustering methods, including *k*-means, spectral clustering, and hierarchical clustering, with the F-measure of 0.935 on the LFW dataset.

Liu et al. [17] modified FaceNet and constructed a liveness detection model by attaching the Kinect infrared (IR) sensor to avoid face spoofing attacks while simultaneously performing face recognition. They mainly introduced a classification method—support vector machine (SVM) following FaceNet—to determine the similarity of the recognized face and various other faces. Their approach was able to effectively avoid face spoofing while recognizing a face and for authentication purposes. However, the structure of FaceNet was not reduced in their work, and its computational burden was still heavy and therefore not suitable for deployment in embedded devices. For most similar studies using multiple spectral images, such as visible and IR images, image registration is the main issue, as discussed in [18,19]. Images from multiple sensors must be fused before applying face recognition. Additionally, a threshold needs to be predefined for distinguishing the recognized face from other faces in the database on the basis of the predicted similarity values. The authors also built an unknown category to tackle the issue of false recognition. The obtained results showed a tremendous reduction in false recognition rate, and the model with liveness detection and face recognition was able to be deployed for identity authentication. Lee et al. [20] constructed a lightweight and computationally efficient model to perform face recognition for a stand-alone access control system. The proposed model was based on the framework composed of the local binary pattern (LBP) and the AdaBoost classifier. The Gabor-LBP histogram was modified by applying Gaussian derivative filters as alternatives to Gabor wavelets to extract facial features. In addition, AdaBoost was used to perform a rapid face and eye detection with the model invariant to illumination changes. The results showed an accuracy of 97.27% and 99.06% on the E-face and the XM2VTS datasets, respectively.

Three-dimensional (3D) face recognition is another branch of face recognition techniques for overcoming issues arising from ambient light, background, and shooting angle. Supported by auxiliary 3D face images and 3D imaging technologies, more accurate results can be obtained [21,22,23,24]. However, the requirements of computational power and equipment increase heavily and become highly dominant. To achieve a lightweight architecture while retaining efficient and highly accurate face recognition, MobiFace [25] and ShuffleFaceNet [26] have recently been proposed. Their accuracy is maintained or even improved with the proposed lighter structures. By doing so, the number of parameters in their neural networks was significantly reduced. However, the development of both networks was still carried out with no assumption of hardware limitations, not to mention the hardware supportability into consideration.

**Figure 2:**
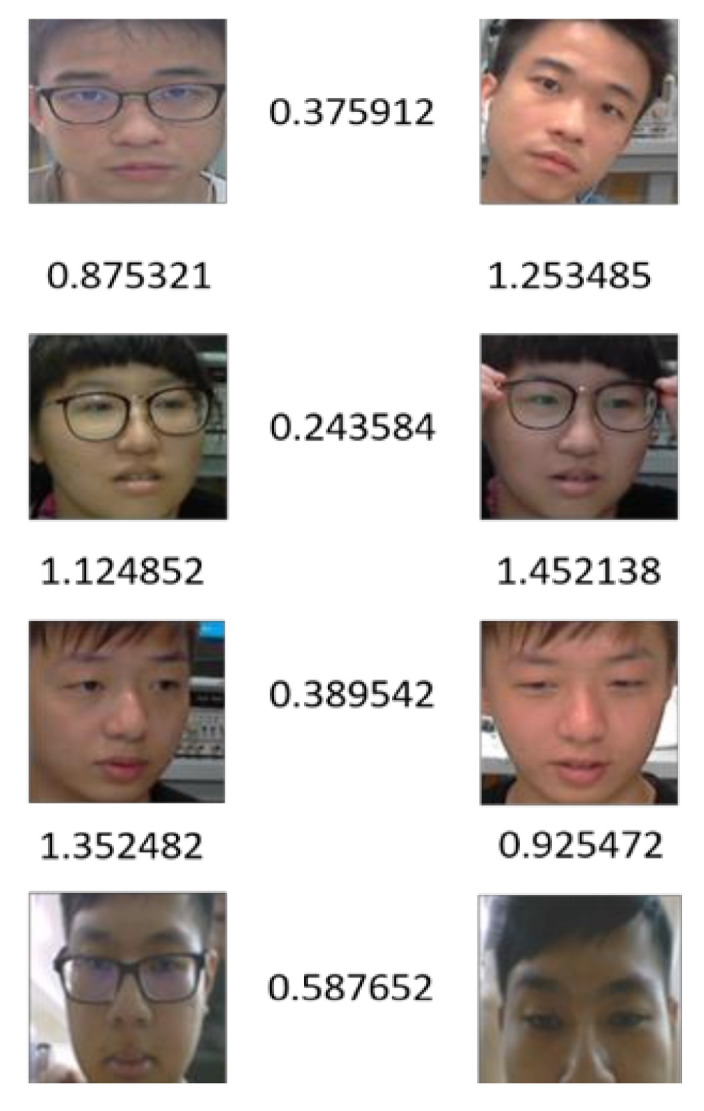
Face recognition using FN13 with variation in terms of lighting and postures. The numerical number between the faces represents the similarity of two images where the score ‘zero’ is considered to represent two distinct people.

Although several face recognition models based on deep learning approaches, as investigated above, have already achieved over 99% accuracy, their architectures make them less than ideal and unsuitable for embedded devices with the constraints of limited hardware resources. Despite the dramatic performance of DL and CNN, most embedded devices cannot support their application while retaining low latency, low power usage, and high precision within the computational resource constraints. To further make face recognition available and implementable on the embedded devices with only grayscale images, a lightweight learning network conceptualized on FaceNet, called FN13, is proposed in this study according to the main hardware limitations, while deploying deep learning in an embedded target. On the basis of the project cooperation with Holtek Semiconductor, which is a leading professional IC design house in Taiwan, the proposed FN13 model can be deployed in a fully integrated device (HT82V82) as the embedded system featuring AI computing for facial recognition applications [27]. To comply with the development constraints for making FN13 work in the integrated HT82V82 package, the first limitation is the constraints on convolutional layers. The second is the constraints on the pooling layer. Last but not least, the limitation of using padding for all layers is considered. It is also worth mentioning that the proposed FN13 model can diminish the large number of parameters required by FaceNet, and very little sacrifice of accuracy on the dataset collected from Labeled Faces in the Wild database (LFW) [28], which was used for evaluating the performance of the proposed face recognition scheme. By using one-shot learning without the need for a retraining process, FN13 can effectively distinguish faces of various identities even if the postures and the portions of the face in the images vary. Prior to detailing our proposed method, Figure 2 depicts the considerable variations in terms of pose and lighting conditions possible when recognizing a face, and shows the feasible recognition ability of FN13.

## 3. Proposed Method

**Figure 3:**
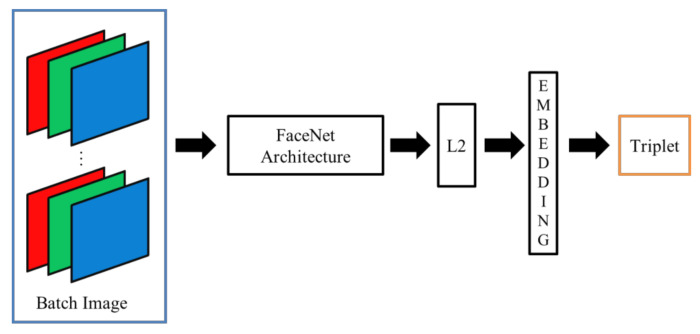
Block diagram of FaceNet architecture.

**Figure 4:**
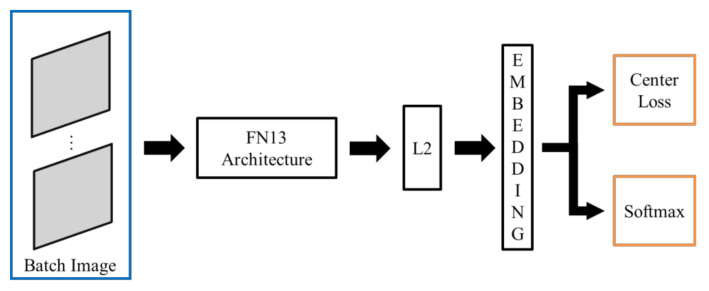
Block diagram of FN13 architecture using center loss function.

Due to the hardware limitations of devices with resource constraints, FN13 is proposed as a lighter framework of FaceNet. Specifically, unlike MobiFace, ShuffleFaceNet, and many other lightweight deep neural networks, as investigated in related works, FN13 is proposed for deployment in a real-time integrated device, HT82V82, with constrained hardware resources. As described in the following sections, the constraints involve the convolution, pooling, and padding layers. Additionally, as depicted in Figure 3, the color channel input of face thumbnails is required for use in the block diagram of FaceNet and other lightweight deep neural networks. However, the computation and memory of the HT82V82 are not sufficient for the state-of-the-art DL and CNN used in color face recognition. With only dual-core DSP processors, the HT82V82 needs an additional hardware engine in one integrated package to accelerate image processing and AI computing. Thus, to confront the computational and storage demands in terms of image processing for the target device HT82V82, FN13 takes greyscale images as its input and instead uses center loss as its loss function for training the network. The architecture of FN13 is shown in Figure 4. To construct this framework, a list of constraints in practical implementation are addressed in the following sections, and thus, face recognition can be implemented under such hardware limitations.

### 3.1. Facial Image Preprocessing

To meet the computational and storage demands in terms of image processing, FN13 needs to use grayscale images for facial recognition. The conventional way to generate a grayscale image is to take the average intensity from the three color bands as follows:

(1) $$Grayscale1=\left(R+G+B\right)/3$$

where R, G, and B represent the red, green, and blue band, respectively. However, this transformation will not fit real case scenarios, since the three color bands do not contribute evenly to the image. Instead, the coverage of each color band is quite varied, with the red color covering a wider range of wavelengths than the other two colors. Additionally, human eyes are more sensitive to the green color, which is composed of a shorter range of wavelengths than red and blue. Thus, it is more appropriate to transform color images with some weights that would increase the fraction of green color and decrease the fraction of red color. Therefore, FN13 can use a weighted transformation to generate good quality grayscale images, as follows:

(2) Grayscale2=0.2989×R+0.5870×G+0.1140×B

Furthermore, in order to increase the variety of samples in the training stage, the training images are rotated randomly. During rotation, images must be resampled and some interpolation methods should be applied to generate images with few interpolation artifacts. In this study, the bicubic interpolation method is applied prior to training to build a robust face recognition model. Unlike the bilinear method, which only takes four pixels into account at a time, the bicubic method regenerates 16 pixels by taking the four corners of a unit square along with various image directions. Under such circumstances, the solution would form a bicubic surface that can be used to generate smoother rotated images with fewer artifacts. Suppose that the function h represents the image, with $h_{x},h_{y},$ and $h_{xy}$ representing three derivative images of h that can be calculated by taking derivatives, respectively, in the three directions, x, y, and xy. Additionally, they are assumed to be known at the four corners, (0,0), (0,1), (1,0), and (1,1), of a unit square. Let $f\left(x,y\right)$ be the interpolated surface that can be written as:

(3) $$f\left(x,y\right)=\sum_{i=0}^{3}\sum_{j=0}^{3}a_{ij}x^{i}y^{j}$$

By substituting the four corners into (3) and solving the system formed by using the resulting equations, better, smoother, rotated images with fewer artifacts can be obtained for the training stage of the FN13 model.

### 3.2. Hardware Limitation Issues

To make a deep learning recognition model available and implementable on embedded devices, the model must be designed to fit the hardware limitations of the given devices. Here, three main types of hardware resource limitations on the target device HT82V82 are considered, which can be associated with the primary components that form CNN. The first limitation is the constraints on the convolutional layer. Only a size of 3 × 3 or 5 × 5 kernels can be accepted in the convolutional layer, with an input size no less than 5 × 5. The second limitation is concerning the pooling layer, due to its low data reusability. In the supported library of software kernels on HT82V82, only max pooling is available, with its kernel being either 2 × 2 or 4 × 4. Third, no padding is available for all layers. The details of the constraints are listed in Table 1.

### 3.3. Convolutional Network Architecture

**Figure 5:**
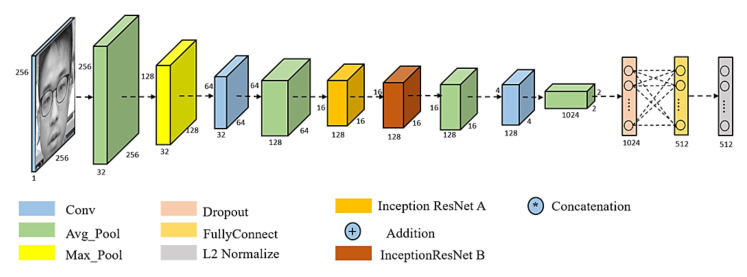
The framework of FN13 model.

The proposed FN13 model is composed of a batch input layer that receives grayscale images followed by CNN with L2-normalization applied as the distance measuring. FN13 learns the best face representation as face embedding for each face class via center loss along with softmax loss. Figure 5 shows the architecture of the proposed FN13 model.

Table 2 tabulates the parameter settings of the proposed FN13 model layer by layer. The proposed network consists of a grayscale image input layer with a size of 256 × 256, a convolutional layer with a size of 3 × 3 kernels, no paddings, and a pooling layer with a size of 2 × 2 or 4 × 4. The resulting FN13 drastically reduces the computational loading by lowering the number of parameters from the 20.95 M used in FaceNet (Inception-ResNet-v1) to 7.76 M, while still meeting the requirements of the aforementioned hardware resource constraints of HT82V82. In addition to reducing the number of parameters, the pooling operation is used as an alternative to the stride operation following all the convolutions. According to the experimental results obtained in Section 4, only a small drop in terms of accuracy is shown when discarding the stride operation. Notice that through the feasible reduction of the number of parameters, FN13 can be deployed in embedded devices due to the resulting lightweight structure. The reduced number of parameters can save storage space in the device and increase its computing efficiency. Speaking of FaceNet, however, there is no way to reduce the parameters without modifying its network structure, which makes it not suitable for embedded devices.

**Figure 6:**
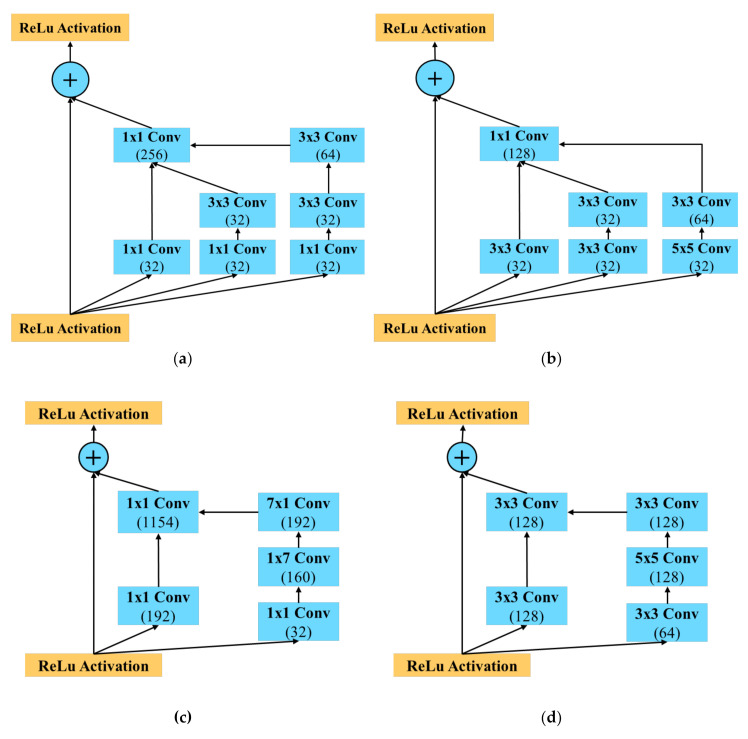
Inception structure modification: (**a**) Inception-ResNet-A structure with two 3 × 3 convolutions, (**b**) modified structure based on Inception-ResNet-A, (**c**) Inception-ResNet-B structure with 1 × 7 and 7 × 1 convolutions, (**d**) modified structure based on Inception-ResNet-B.

The referenced FaceNet (Inception-ResNet-v1) is constructed based on the idea of inception, using 1 × 1 convolution and pooling layers in parallel to remove possible redundant parameters. In order to achieve the goal of making the recognition model lighter, the concept of inception is kept. However, due to the limitations in kernel size that can be used, 1 × 1 convolution has to be replaced. Figure 6 shows the example of modifying the inception structure based on different sizes of the convolutional kernel. The modified structure will have the same width as the one it is inherited from. As such, FN13 can simply take 3 × 3 and 5 × 5 kernels instead of a 1 × 1 kernel in the Inception ResNet. Additionally, FN13 modifies the online mining method used in FaceNet [2] to generate center loss instead of non-matching face pairing. Thereby, an output with 512-D embedding face representation is obtained.

### 3.4. Center Loss

**Figure 7:**
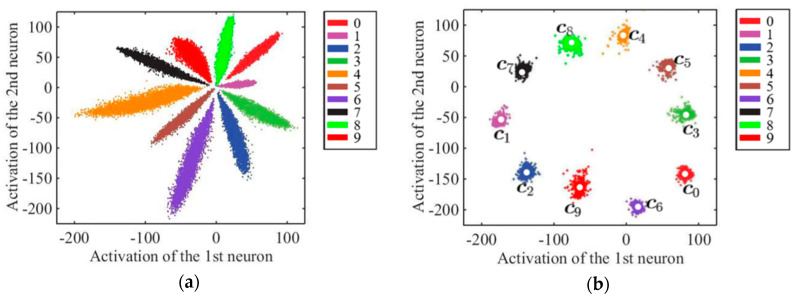
Example comparison of features learned under the supervision of softmax loss function with/without center loss introduced to the model. (**a**) Without center loss, and (**b**) with center loss; points with different colors represent features for various classes.

FN13 uses center loss [29] along with softmax loss as its loss function for training the face recognition model. With the application of center loss, FN13 learns the center for each face class according to the face embeddings derived from each class as features. Simultaneously, during the training stage, the center for each class is updated, while the distance between within-class features and the center is minimized. Under the joint supervision of center loss and softmax loss, distances between within-class features and the class centers are minimized as the distances between different classes are maximized. In other words, the softmax loss keeps the different face classes apart and the center loss keeps the features within the same class closer to its center. As a consequence, not only are differences in within-class features increased, but the variations in between-class features are also reduced. Figure 7 depicts features in two-dimensional space as an example to illustrate the learning power of using center loss jointly with softmax loss.

The center loss characterizes the within-class variation by defining

(4) $$L_{c}=\frac{1}{2}\sum_{i=1}^{m}{\left\|x_{i}-c_{y_{i}}\right\|}_{2}^{2},$$

where $x_{i}$ denotes the extracted feature, $c_{y_{i}}$ denotes the center of class $y_{i}$, and m denotes the batch size. To make the model work efficiently, the centers must be updated only depending on the current batches with their corresponding classes. Therefore, the center can be updated by taking the gradient of $L_{c}$ with respect to $x_{i}$ to derive the update function $\Delta c_{j}$ for class $c_{j}$ as

(5) $$\frac{\partial L_{c}}{\partial x_{i}}=x_{i}-c_{y_{i}}$$

(6) $$\Delta c_{j}=\frac{\sum_{i=1}^{m}\delta \left(y_{i}=j\right)\cdot \left(c_{j}-x_{i}\right)}{1+\sum_{i=1}^{m}\delta \left(y_{i}=j\right)}$$

where $\delta \left(\cdot \right)$ is the condition function that will be satisfied as $y_{i}=j$. To make center loss collaborate with softmax loss, the objective loss function is re-formulated by

(7) $$L=L_{s}+\lambda L_{C}$$

With

(8) $$L_{S}=-\sum_{i=1}^{m}\sum_{j=1}^{n}\delta \left(y_{i}=j\right)\log\frac{e^{W^{T}x_{i}+b}}{\sum_{l=1}^{n}e^{W^{T}x_{l}+b}},$$

where λ denotes the weighting influence of the class-wise center loss, *n* is the number of individuals stored in the database, ***W*** is the corresponding weight in FN13, and *b* represents the bias in the model.

### 3.5. One-Shot Learning

In general, a deep CNN model requires a huge dataset to train a model with high accuracy. However, this is not practical in face recognition, because, usually, not many images can be collected for one individual. One-shot learning [30] sheds light on such an issue. It allows a model to be trained using only a few samples of the subjects, without being retrained when introducing new subjects. To implement this idea, face embedding is the key. One-shot learning learns a similarity function to measure the similarity of the input image with the stored images over their corresponding embeddings. Similar images will have higher similarity, whereas non-similar images will have a higher loss. Once a new sample is presented, the trained network will generate its corresponding embedding and calculate the similarity to all stored images. For further recognizing this newly presented sample, the trained network does not need to be retrained, but only to store its embedding along with its labeling.

## 4. Experiments

The recognition models are implemented with a device using a general PC development setup and Microsoft Windows 10 operating system. To compare the performance for FN13 and FaceNet on various computing environments, the experimental results are conducted on CPU-based device with/without GPU supported.

### 4.1. LFW Dataset

**Figure 8:**
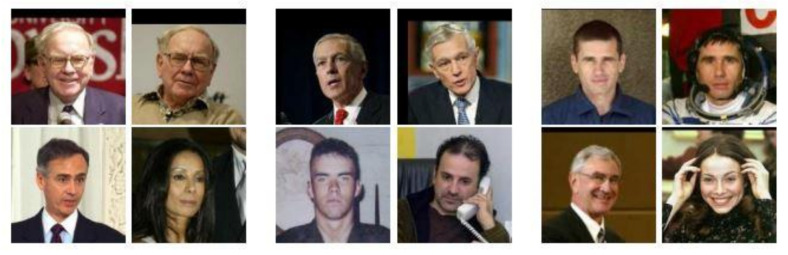
Image examples selected from the LFW dataset.

The dataset used to conduct experiments on the proposed FN13 was collected from the LFW. LFW was designed for unconstrained face recognition studies, with more than 13 thousand name-labeled face images, including 1680 individuals having more than one distinct photo, all collected from the web [28]. In order to test FN13′s capability for face recognition, the entire dataset of 13 thousand photos was taken as the test data in the experiments. Figure 8 shows a few image examples from the LFW dataset. The FN13 model was pre-trained using the CASIA-WebFace [31], crawled from the Internet by the Institute of Automation from Chinese Academic Science, to get FN13 to learn the best feature representation of each face photo as embedding. Thereby, FN13 can generate the feature embedding corresponding to each test data.

### 4.2. Performance Evaluation

**Figure 9:**
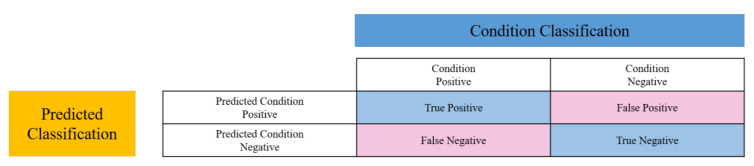
The confusion matrix for the face recognition system for evaluating overall performance.

Figure 9 depicts the confusion matrix for a binary classifier, which is the face recognition system in this manuscript, to evaluate the overall performance. All subjects are categorized into two groups, namely, condition positive and condition negative. The condition positive group consists of all sample images belonging to a subject that is to be identified, while the condition negative group includes other sample images. Accordingly, four compound conditions, including true positives (TP), true negatives (TN), false positives (FP), and false negatives (FN), can be defined to analyze the correctness and robustness of the proposed FN13 model. To evaluate the performance of FN13 and compare it with other state-of-the-art face recognition models, the model accuracy, precision, recall, and F_1_-score are computed.

Precision and recall are two measures for indicating the true positive rate from all predicted positives and true predicted rates of all positive samples, respectively. The higher the precision, the higher the degree of correctness for positive samples. Similarly, the higher the recall, the stronger the ability of the recognition model to distinguish negative samples from positives. In addition, the F_1_ score measures the harmonic mean of the precision and recall to take both measures into account in order to deal with the issue of uneven class samples.

Table 3 summarizes the performance comparison among some typical CNN-based recognition models, including computational complexity in FLOPs, the number of parameters, and the memory storage of models. As can be seen, FN13 is superior to the others in terms of complexity in FLOPs. This is due to the fact that FN13 is under the constraint of limited hardware support in computing resources, where no more complicated arithmetic is allowed. As a consequence, FN13 has a slightly larger model size compared to FaceNet, since there exists a trade-off between reducing complexity and reducing the size of the model.

### 4.3. Experimental Results

Firstly, the LFW dataset was used to evaluate the proposed FN13 model with ten-fold cross-validation used to select an appropriate L2-normalization distance threshold for discrimination among the different classes based on FN13-generated face embeddings. The LFW dataset was split into 10 folds for conducting experiments, including 9 training folds and one test fold, and 0.6 was chosen as the optimal threshold for all tests. The performance of FN13 was compared to FaceNet, and the results are listed in Table 4. To overcome the limitations for having constrained hardware resources on embedded devices, FN13 exhibits a lighter structure with fewer layers and fewer parameters than FaceNet does, which results in a higher false acceptance rate (FAR). Nevertheless, only using grayscale images, the overall accuracy for recognizing known faces remains at a high level, at 96.65% correctness.

To compare the time performance of FN13 to FaceNet, both models were implemented in computing environments with and without a Graphical Processing Unit (GPU)-accelerated computing device to test and conduct the experiment using 300 input images. As shown in Table 5, with the lighter structure, FN13 took less time than FaceNet to recognize a face under both computing environments and showed a great improvement in time when using a GPU for acceleration. FN13 was further improved to enhance recognition ability by introducing more image samples associated with one individual subject. For instance, FN13 became more accurate when taking not just one sample, but three samples into account, while not sacrificing much in terms of time.

Once the device captures a face image, the corresponding 512-D facial descriptor is generated via FN13. To further identify the identity of the face, Mahalanobis distances between the generated facial descriptor and the stored multi-sample embeddings for each identity class are then calculated. The face image should be assigned to the identity class with the shortest distance among all classes. However, the face image could represent an unregistered face. Thus, to avoid such misidentification, a threshold is preset as a criterion. If the minimum distance between the facial descriptor and stored embeddings is less than the threshold, the face image would be assigned to the ID belonging to the identity class with the shortest distance from any of its multi-samples. Otherwise, the recognition system would deny access to this unrecognized identity. Algorithm 1 describes the steps to perform embedding matching with multiple samples.

**Algorithm 1.** Embedding Matching with Multiple Samples**Input:** A 512-D facial descriptor (embedding extracted by FN13): $x={\left[x_{1},x_{2},\ldots ,x_{512}\right]}^{T}$. **Output:** Recognized ID: id∈ℝ
Get the stored facial embedding set $E_{j}=\left\{e_{1,j},e_{2,j},\ldots ,e_{i,j}\right\}$ of identity class j, where i represents the number of sample embedding in identity class j.Calculate the Mahalanobis distance (M-distance) between **x** and each facial embedding $e_{i,j}$ in $E_{j}$ as $D_{k}=M_{dist}\left(e_{k,j},x\right)$.Update the minimum distance $D_{min}$ and $id_{temp}$ if exists any $D_{k}<D_{min}$. That is, $D_{min}=\min\left\{D_{k}|k=1,2,\ldots ,i\right\}$ and $id_{temp}=j$.Let j=j+1. Repeat 1–3 until no more identity classes are found.Given a preset threshold thrshld. If $D_{\min}<\mathrm{thrshld}$, then assign the output id by $id_{\mathrm{temp}}$. Otherwise, the output id would be set to −1 as an unrecognized identity.


Under such circumstances, FN13 obtained 98.41% accuracy, which is about 2% more accurate than using one sample, and with only a 0.00097 s delay. This is due to the fact that the model generates the corresponding embedding for the input image only once. In addition, FN13 using more samples for recognizing can accommodate itself to the changed grayscale input, which is less informative than the color images used by FaceNet, without losing accuracy. This makes FN13 superior to FaceNet. With respect to the size of the input and the size of embedding used by both recognition approaches, it can be found notably that FN13 requires a much smaller amount than FaceNet. This also demonstrates the possible transplantation for the FN13 model for implementation in real-time onboard processing.

**Figure 10:**
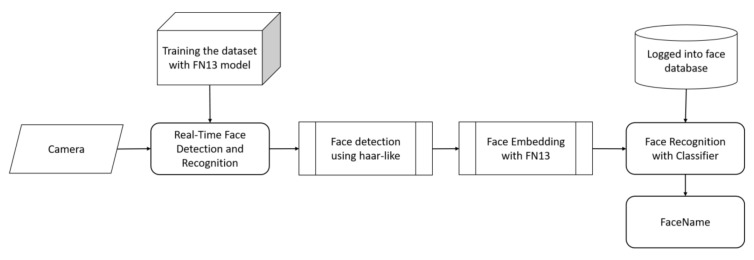
Processing flowchart with FN13 on a real-time face recognition embedded system.

**Figure 11:**
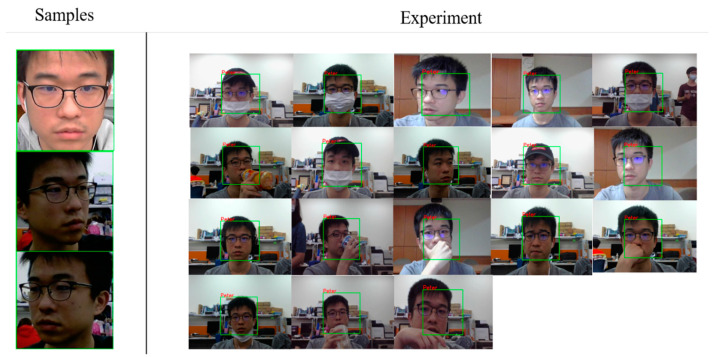
Real-time camera recognition of a known face under various conditions (with three samples).

For recognizing a face in a real-time face recognition system, the process consists of four stages with a captured facial image being loaded into the system, including face detection, embedding generation, face recognition, and output response. For detecting a face, hair-like features that capture the structural similarities within faces can work effectively. Thus, the detected face can be further recognized with the face embedding generated by FN13. The recognition result will be stored for further analysis if a known face exists in the input image, or an alert may be sent if it detected a possible intrusion. Figure 10 illustrates the real-time processing flowchart with FN13 implemented in a vision-based human face recognition system. From the acquisition of an optical camera in real-time, the raw input for our system is the face image of a user. Figure 11 shows an example of recognizing a known face using three samples via FN13 under various conditions, including the variation in terms of occlusion, lighting, and posture changes.

In addition to the performance evaluation for real-time recognition, generated feature embeddings can be inspected via various classifiers as mentioned in [36]. The features are obtained by the same CNN structure with softmax and center loss function. The classification results via various classifiers with the generated features are then compared with FN13 using the same loss functions. Table 6 presents the performance comparison of FN13 with respect to various typical classifiers including eXtreme Boosting Gradient (XGBoost) [37], Light Gradient Boosted Machine (LightGBM) [38], and Gradient Boosting Decision Tree (GBDT) [39], on LFW dataset. As can be observed, FN13-generated face embeddings provide good face representations and make most classifiers available to distinguish different faces effectively; thereby, FN13 outperforms the other models.

**Figure 12:**
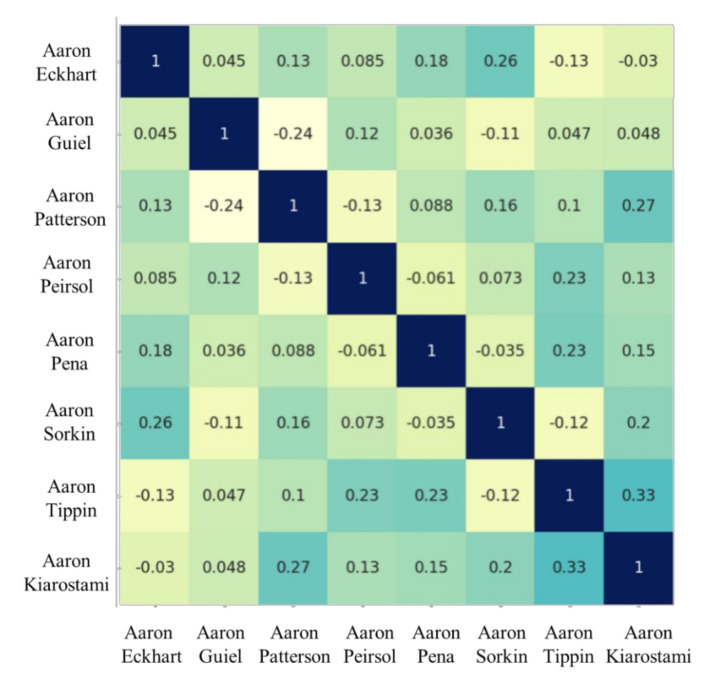
The matrix of similarity examples for the LFW test set with FN13-generated face embedding corresponding to each sample image.

Figure 12 shows the similarity between faces via Pearson product–moment correlation with values ranging within [−1, 1]. The vertical and horizontal axes are formed by the first eight sample images listed in the LFW dataset. The correlation between two samples is calculated based on their corresponding face embeddings so that all the values in the diagonal line are those with the darkest background color, representing two identical sample images. The darker the background color (the larger the value) is, the greater the similarity the image pair possesses, and zero correlation suggests that the two images share nothing in common within their face embeddings. Despite the diagonal line in Figure 12, correlations for image pairs of two different samples are within the range [−0.24, 0.33]. This implies that the FN13-generated face embeddings can specify and separate different identities into face classes easily. Consequently, the face recognition system can achieve a good true negative rate, while the overall accuracy can be maintained.

### 4.4. Discussion

**Figure 13:**
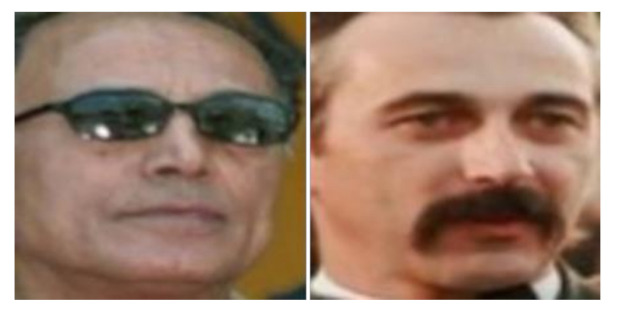
Image pair sharing the maximum correlation coefficient in the example of the LFW.

To further analyze the correlation among different identities, the image pair sharing a correlation coefficient of 0.33 in the example of the LFW is discussed. As can be seen in Figure 13, the image with a man wearing sunglasses on the left would have a larger similarity value with the image of another man with a mustache. This may be a result of significant facial features being covered and disguised by accessories or facial hair. As a consequence, the facial pattern recognition model would have difficulty extracting facial descriptors, such as the shape and depth of eye contour, the distance between eyes, or even eyebrows, which means that the model has to rely on other descriptors, lowering its discrimination ability. Additionally, it turns out that the Euclidean distance measure used by FaceNet cannot distinguish the patterns among various classes due to dependencies between generated embedding vectors. The Mahalanobis distance measure was then considered as an alternative to get rid of the abovementioned relations among each signature via the following equation:

(9) $$D^{2}={\left(x-\mu \right)}^{T}C\left(x-\mu \right)$$

where *D* is the Mahalanobis distance, *x* is the vector of the facial descriptor, *μ* represents the mean vector of the facial descriptor, and *C* shows the covariance of vectors.

**Figure 14:**
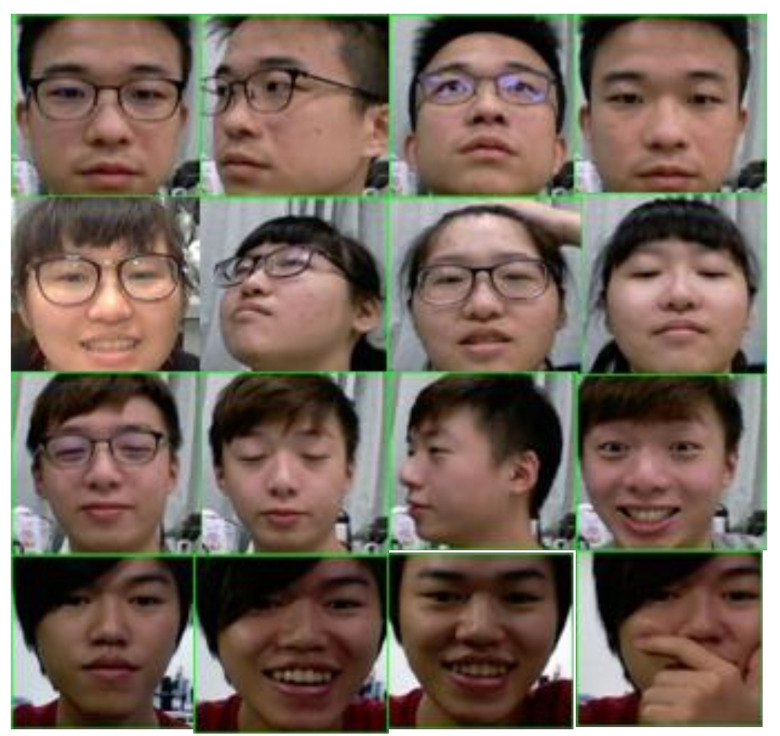
A collection from a self-collected, non-public dataset.

**Figure 15:**
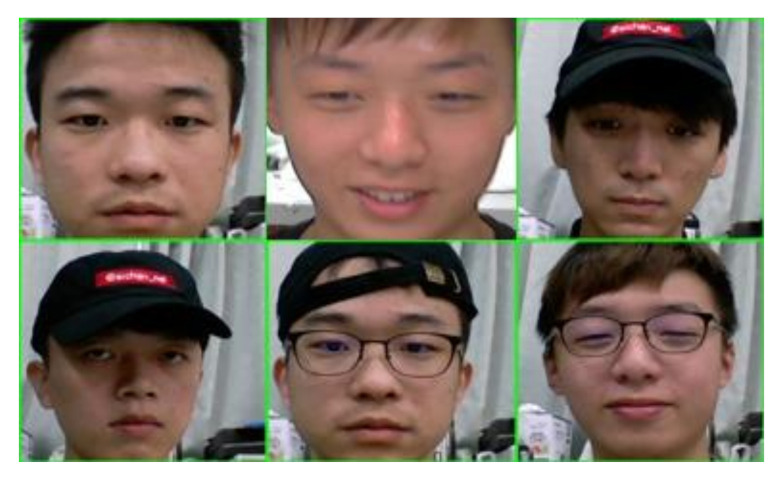
Example of falsely accepted face images.

**Figure 16:**
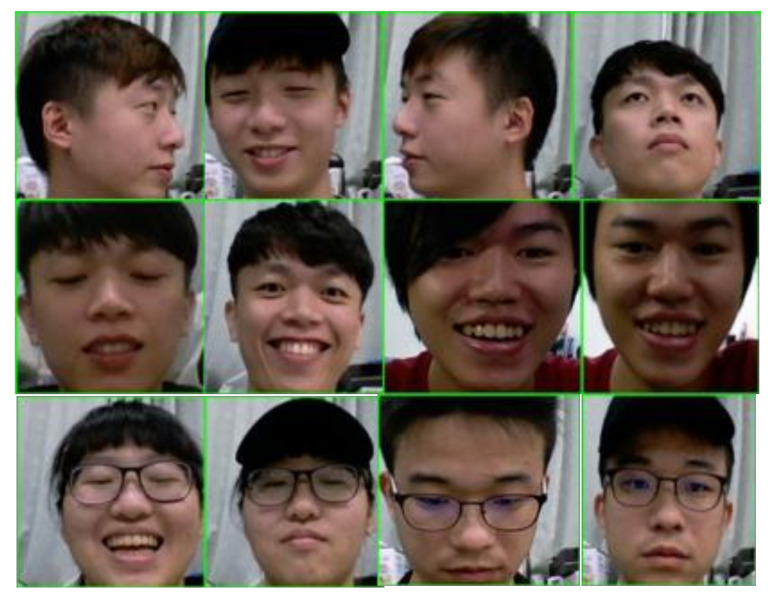
Example of falsely rejected face images.

**Figure 17:**
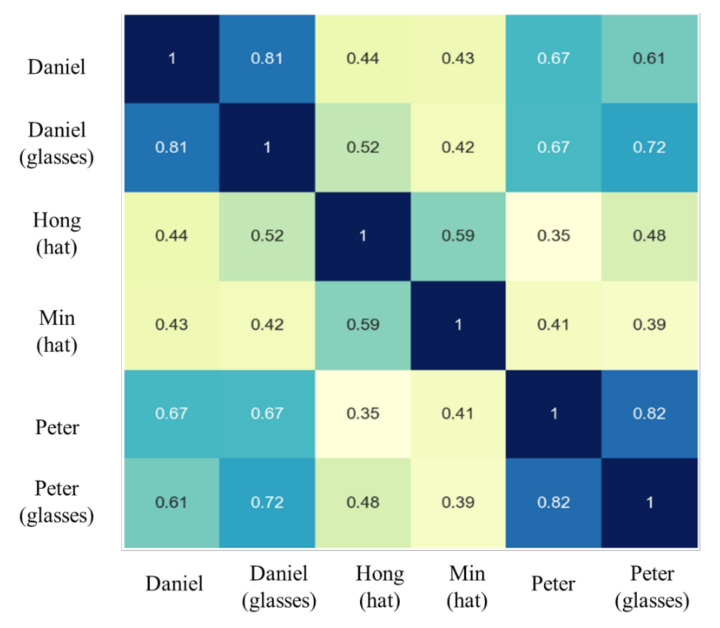
The matrix of similarity for the falsely accepted face images shown in Figure 16.

Next, a self-collected, non-public dataset was used to promote and validate FN13. Figure 14 shows a collection of images that were tested and recognized by FN13. To test the model capability and observe possible conditions that cause error recognition, subjects were asked to cover their usual appearance or to intentionally avoid facing the camera. The experiments show two types of errors. The first type is the false acceptance type, where the recognition model misidentified one subject as another registered subject. The other type is the false rejection type, where a registered subject could not be identified. Figure 15 and Figure 16 show some case images that resulted in false acceptance and false rejection, respectively. It can be observed from the images that the error identification occurred while the test subjects were wearing some accessories or had their vital facial signatures disguised. Additionally, it was found that the signatures surrounding eyes are most decisive. Eye closing, shades overlaying eye area, and hair covering eyes all caused the model to fail. As the corresponding similarity analysis shows in Figure 17 for those false acceptance cases, it can be found that the correlation among them was higher than the normal situation. In other words, the similarity becomes higher if two identities intentionally wear the same accessories. Despite this observation, it is worthy to note that an individual with or without accessory would still contribute the highest similarity, for instance, Daniel with or without glasses, and Peter with or without glasses. This intriguing finding again validates that the correctness and robustness of our proposed model can be improved by adding a few more samples for recognizing one identity.

## 5. Conclusions

In this paper, FN13, a novel lightweight face recognition model based on FaceNet, was proposed to overcome hardware limitations that may occur when the recognition model is implemented in an embedded target. To achieve real-world deployment of deep learning in the target device HT82V82, developed by Holtek, FN13 was designed in consideration of the constrained resources required in the embedded environment, such as the constraints of size input, the constraints of specific sizes that can be used in deep learning models, and the constraints of supported operations. In contrast to FaceNet, FN13 directly trains its output as a compact 512-D embedding and uses the center loss function for training. Through center loss, we can effectively lessen the distance between subjects in the same category and enlarge those within different categories. Center loss enables FN13 to achieve better balance and performance for recognizing faces with constrained hardware resources.

With its light structure and computational efficiency, FN13 can also be implemented in a real-time camera surveillance system to track, identify, and monitor subjects. Based on the proposed method, the theoretical design principles of deep learning are clarified to achieve more efficient simplification and robustness in the execution of face recognition algorithms. As shown in the experiments, FN13 generates a good representation of faces in sample images via face embeddings, and it achieves great performance, while the size and the parameters used to construct the face recognition model are significantly reduced.

## Figures and Tables

**Table 1:** The limitation of hardware resource constraints for implementing the CNN-based method.

Type of Network Layer	Size of Kernel	Size of Input	Stride	Padding
Convolutional	3×3 5×5	≥5×5	1	N/A
Max pooling	2×2 4×4	4N×4N	2 or 4	N/A
	**Max Input Layers**	**Max Hidden Layer**	**Max Output Layer**	**Number of Batch Normalization**
**Overall Structure**	4096	1024	1024	2

**Table 2:** Layer information of the FN13 model designed based on the hardware limitations with the use of *L*_2_-normalization distance.

TYPE	INPUT SIZE	OUTPUT SIZE	KERNEL SIZE	**#PARAMETERS**
Conv1	256×256×1	256×256×32	3×3×32	320
Avg Pool	256×256×32	128×128×32	2×2×32	0
Max Pool	256×256×32	64×64×32	2×2×32	0
Conv2	64×64×32	64×64×128	3×3×128	37 K
Avg Pool	64×64×128	16×16×128	4×4×128	0
Inception-ResNet-A	16×16×128	16×16×128		3.3 M
Inception-ResNet-B	16×16×128	16×16×128		4.3 M
Avg Pool	16×16×128	4×4×128	4×4×128	0
Conv3	4×4×128	2×2×1024	3×3×1024	1.2 M
Avg Pool	2×2×1024	1×1024	2×2×1024	0
Dropout	1×1024	1×1024		0
FC	1024	512		131 K
L2 norm	512	512		0
**Total**				7.76 M

**Table 3:** The performance comparison between the proposed FN13 and other typical CNN models.

Recognition Model	Complexity in FLOPs	Parameters (M)	Model Size (MB)
VGG-Face [15]	15.5 G	138	526
Light CNN-29 [32]	3.9 G	12.6	125
MobileFaceNet [33]	0.44 G	1.0	4
MobiFace [25]	-	-	9.3
ShuffleFaceNet 1.5x [26]	0.58 G	2.6	10.5
PolyFace [34]	24.04 G	128.7	491
PolyFace+QAN++ [34]	24.12 G	128.3	490
Inception-ResNet v1 [35]	2.97G	20.95	91.3
FaceNet [2]	1.6 B	7.5	30
FN13	0.18 G	7.76	57.3

**Table 4:** The performance comparison between the proposed FN13 and FaceNet with leave-one-out validation.

Model	FAR	Input Image Size
FN13	0.0064	256 × 256 × 1
FaceNet	0.001	160 × 160 × 3

**Table 5:** The performance comparison in time on different platforms.

Model		Accuracy	Execution Time (Unit: Second)	
			CPU i7-2600	GPU 1080Ti
FN13	(1 sample)	96.65%	0.07759	0.00803
	(3 samples)	98.41%	0.07929	0.00810
FaceNet		99.65%	0.08192	0.02510

**Table 6:** The performance of the proposed FN13 compared to various classifiers on LFW dataset.

MODEL	RECALL	PRECISION	F1 SCORE	ACCURACY
XGBoost	0.8039	0.8199	0.8089	83.10%
LightGBM	0.7981	0.8043	0.8004	81.69%
GBDT	0.8211	0.8132	0.816	83.10%
FN13	0.9872	0.9467	0.9765	96.65%
